# The first released available genome of the common ice plant ( *Mesembryanthemum crystallinum* L.) extended the research region on salt tolerance, C3-CAM photosynthetic conversion, and halophism

**DOI:** 10.12688/f1000research.129958.1

**Published:** 2023-04-27

**Authors:** Ryoma Sato, Yuri Kondo, Sakae Agarie

**Affiliations:** 1 Graduate school of Bioresource and Bioenvironmental Sciences, Kyushu University, 744 Motooka Nishi-ku Fukuoka, 819-0395, Japan; 2 Faculty of Agriculture, Kyushu University, 744 Motooka Nishi-ku Fukuoka, 819-0395, Japan

**Keywords:** common ice plant, genome release, halophilism, salt-tolerance, salinity, photosynthesis

## Abstract

**Background:** The common ice plant ( *Mesembryanthemum crystallinum* L.) is an annual herb belonging to the genus Mesembryanthemum of the family Aizoaceae, native to Southern Africa.

**Methods:** We performed shotgun genome paired-end sequencing using the Illumina platform to determine the genome sequence of the ice plants. We assembled the whole genome sequences using the genome assembler “ALGA” and “Redundans”, then released them as available genomic information. Finally, we mainly estimated the potential genomic function by the homology search method.

**Results:** A draft genome was generated with a total length of 286 Mb corresponding to 79.2% of the estimated genome size (361 Mb), consisting of 49,782 contigs. It encompassed 93.49% of the genes of terrestrial higher plants, 99.5% of the ice plant transcriptome, and 100% of known DNA sequences. In addition, 110.9 Mb (38.8%) of repetitive sequences and untranslated regions, 971 tRNA, and 100 miRNA loci were identified, and their effects on stress tolerance and photosynthesis were investigated. Molecular phylogenetic analysis based on ribosomal DNA among 26 kinds of plant species revealed genetic similarity between the ice plant and poplar, which have salt tolerance. Overall, 35,702 protein-coding regions were identified in the genome, of which 56.05% to 82.59% were annotated and submitted to domain searches and gene ontology (GO) analyses, which found that eighteen GO terms stood out among five plant species. These terms were related to biological defense, growth, reproduction, transcription, post-transcription, and intermembrane transportation, regarded as one of the fundamental results of using the utilized ice plant genome.

**Conclusions:** The information that we characterized is useful for elucidation of the mechanism of growth promotion under salinity and reversible conversion of the photosynthetic type from C3 to Crassulacean Acid Metabolism (CAM).

## Introduction

Soil salinity is one of the most detrimental abiotic stresses. Osmotic and ionic stresses can lead to decreased plant growth and economic damage, with estimates suggesting that it costs the global economy around $27.3 billion annually in lost crop yields ( Qadir *et al*. 2014). Developing a wide range of strategies for adapting to and mitigating NaCl stress is required to address the negative impacts of salinity. Efficient resource management and crop improvement will help overcome the salinity-induced damages to agricultural production ( Shrivastava and Kumar 2015). *Mesembryanthemum crystallinum* L. or the common ice plant is an annual plant of the family Aizoaceae, native to South Africa. This plant survives in the presence of a high salt concentration, even higher than that of seawater and can accelerate its growth under moderate salinity around 200 mM NaCl, wherein the growth and development of most crops are severely inhibited (halophilism; Agarie 2004). Also, it converts its photosynthetic mode from C _3_ to Crassulacean acid metabolism (CAM) under severe salt stress and drought stress ( Adams *et al.* 1998). For the past half-century, the common ice plant has been frequently used as a model for elucidating the mechanisms of salt stress tolerance and photosynthetic conversion in response to salt and drought stresses.

Recently, the molecular processes underlying these phenomena have been elucidated at the levels of transcription, post-transcription ( Taybi and Cushman 1999; Zhang *et al.* 2021), translation, post-translation ( Forsthoefel *et al.* 1995; Nimmo 2000), specific proteins (tonoplast and plasma-membrane H(+)-ATPases: Vera-Estrella *et al.* 1999; glucose 6-phosphate/phosphate translocator: Kore-eda *et al.* 2013; ions transporter and compatible solute synthase: Tran *et al.* 2020a), mitochondria, and chloroplasts ( Tran *et al.* 2020b; Niewiadomska and Pilarska 2021). Also, high-throughput gene expression profiling has been conducted using expression sequence tags (ESTs) ( Kore-eda *et al.* 2004), microarrays ( Cushman *et al.* 2008), and next generation sequencing (NGS; Oh *et al.* 2015; Tsukagoshi *et al.* 2015; Chiang *et al.* 2016; Kong *et al.* 2020).

In these days, the comparative analysis of genome information focus on CAM related genes on the common ice plants has been reported ( Shen *et al.* 2022), but this genome resource is not easy to use. The poor transparency of genome information causes the delayed elucidation of whole-genome functions of the common ice plants dominating not only photosynthetic conversion systems but also halophilism and salt tolerance. The genomic sequences include protein-coding regions and untranslated regions such as promoters and terminators. MicroRNAs (miRNAs) and long noncoding RNAs (lncRNAs) influence gene expression through affecting mRNA stability and translation efficiency ( Hughes 2006). Information regarding the genome sequences’ biological functions facilitates a comprehensive understanding of the transcriptional regulatory mechanisms of gene expression. Disclosure to researchers around the world is essential for clarifying the responsibilities of the entire genome to NaCl and creating superior cultivars through genome editing and selective breeding.

Short-read sequencing costs less than the long-read sequencing obtained using third and fourth NGS. Several software programs for *de novo* genome assembly for short reads have been developed. The algorithm for genome assembly (ALGA) is the newest assembler, based on an overlapping graphs model, which can generate more accurate results than conventional software using the de Bruijn graphs model ( Swat *et al.* 2021). This achievement can be regarded as a model case of a genome study using NGS short reads, given its level of success.

In this study, we constructed the ice plant genome using easy-to-start applications such as ALGA to accelerate genome analysis. We investigated the characteristics of the genome, clarifying the repetitive sequences, tRNAs, and miRNAs (genomic regions and precursors), and identified gene regions using various software and web tools. This is the first report of whole-genome analysis of the common ice plant. Our results indicate the involvement of translated and untranslated regions in the regulatory processes of salt tolerance and photosynthetic conversion under stress in the ice plant.

## Methods

All the processes involved in this study were archived in protocol.io ( Sato *et al.* 2023a) and were described in Figure S8 ( Sato *et al.* 2023b).

### Plant materials and growth conditions

Seeds of the common ice plant ( *Mesembryanthemum crystallinum*) were personally provided by Dr. John C. Cushman from the University of Nevada and stored under coolness and darkness until use. Originally, wild-type seeds were collected from the plants identified by Dr. Klaus Winter, an expert on the common ice plant, on a coastal cliff at the Mediterranean Sea shore close to Caesarea in Israel (around N32° 29′ 43.4″, E34° 53′ 22.8″) in 1978 ( Winter *et al.* 1978). Three voucher specimens of *M. crystallinum* have been deposited in the Herbarium at the Royal Botanic Gardens Kew (55793.000, K000296094, and K000267571). In this study, our biological materials were recognized as the same plants as those specimens. Experiments, including collecting samples for this study, were conducted in compliance with relevant institutional, national, and international guidelines and laws. The seeds were aseptically sown on a medium for germination containing 4.6 g L ^-1^ MS salt (mixed salts for Murashige-Skoog medium), 30 g L ^-1^ sucrose, 1 mL ^-1^ B5 vitamin ( Gamborg *et al.* 1968), 1 g L ^-1^ nicotinic acid, 1 g L ^-1^ pyridoxine hydrochloride, 10 g L ^-1^ thiamine hydrochlorides, and 100 g L ^-1^ myo-inositol), 0.8% (w/w) agarose, and pH 5.7. The raising of seedlings was performed according to the methods published by Agarie *et al.* (2009). The two-week-old seedlings grown in a growth chamber under 12 h of light and 12 h of darkness at 25 °C were transferred to plastic pots filled with the growth medium soils composed of 50% peat moss, 30% cocopeat, and 20% perlite, specified for the ice plants (Japan Agricultural Cooperatives Ito-Shima, Fukuoka, Japan) and irrigated with a nutrient solution of 1.5 g L ^-1^ OAT House No. 1 and 1.0 g L ^-1^ No. 2 (OAT Agrio Co., Ltd., Tokyo, Japan) in a greenhouse at Kyushu University for five weeks. The plants were treated with the solution including 0.3% (w/w) NaCl for two weeks. Approximately 0.6 g of tissue from each leaf was collected, quickly frozen in liquid nitrogen, and stored at −80 °C.

### DNA extraction, library construction, and sequencing

Total genomic DNA was extracted from the leaf tissue and purified using MagExtractor™-Plant Genome Nucleic Acid Purification Kits (Toyobo Co., Ltd., Shiga, Japan), according to the manufacturer’s instructions. The DNA samples were fragmented by sonication and used to construct short insert paired-end libraries construction using NEBNext ^®^ Ultra™DNA Library Prep Kits for Illumina (New England Biolabs Ltd., Ipswich, MA, USA). Briefly, in the end-repair step, fragmented DNA was phosphorylated at the 5′ end and adenylated at the 3′ end. During the ligation step, full-length circulated adaptor sequences were ligated to the fragments. After adaptor cleavage, purification and size selection were performed. The indexed PCR products were taken to obtain the final sequencing libraries. The mean insert size for paired-end libraries was 300 bp. The paired-end (2×150 bp) sequencing was conducted on an Illumina NovaSeq 6000 platform (Illumina Inc., San Diego, CA, USA).

### Clean read preparation and genome size estimation

The mean insert size was calculated using REAPR (v1.0.18) ( Hunt *et al.* 2013), and raw paired-end sequences were filtered based on the frequency of 21-mer sequences using the program Musket (v1.1) ( Liu *et al.* 2013). The key parameter values were as follows: musket -omulti output -inorder pair1.fastq pair2.fastq. Sequence reads that appeared rarely or abnormally frequently were removed to obtain clean read data. In the corrected reads, unique and duplicate read numbers in the corrected reads were measured using fastqc (v0.11.9) ( Simon 2010). The clean data were used for an estimate of genome size as follows. *K*-mers were counted and exported to histogram files using jellyfish (v2.3) ( Marçais and Kingsford 2011) [key parameter: jellyfish histo reads.jf]. GenomeScope2.0 ( Ranallo-Benavidez *et al.* 2020) corresponding key parameters were applied to calculate the genome sizes using *k*-mers lengths of 21 and 25.

### *De novo* genome assembly and quality evaluation

The reads were assembled using ALGA (v1.0.3; Swat *et al.* 2021) with the default parameter --error-rate = 0.02. long DNA fragments 1 to 10 kb in length were combined, and gaps between them were filled with unknown bases (Ns) using Redundans (v0.14a; Pryszcz and Gabaldón 2016), a software program for scaffolding, with default parameter values. The genome coverage of reads was estimated using Mosdepth program ( Pedersen and Quinlan 2018). The completeness of the assembled genome was evaluated based on the content of orthologs in higher plants, using the benchmarking universal single-copy orthologs (BUSCO) program (v5.0; Manni *et al.* 2021). The lineage dataset was embryophyta_odb10 (creation date: 2020-09-10, number of BUSCOs: 1614). We also searched for core genes in the genome sequences of nine other plant species: *Kewa caespitosa*, *Pharnaceum exiguum*, *Macarthuria australis*, *Solanum chaucha*, *Populus trichocarpa*, *Arabidopsis thaliana*, and *Oryza sativa* using BUSCO. The first three species belong to the same order, Caryophyllales, to which the ice plants belong. Genome information was obtained from the NCBI (see Note 1 “Address to genome information”, Sato *et al.* 2023b). The number of bases, sequences, sequences in several base number ranges, and maximum base length of the final draft genome sequences was calculated using gVolante (v2.0.0) ( Nishimura *et al.* 2017). BLASTN (v2.2.31+; McGinnis and Madden 2004) was used to investigate the number of cDNA sequences identified by transcriptome ( Lim *et al.* 2019), and registered DNA sequences (retrieved from NCBI, last accessed February 2022) were aligned to the final assembled genome sequence.

### Phylogenetic tree creation among multiple plant species using 18S ribosomal DNA sequences

The 18S ribosomal genes were extracted using barrnap (v0.9; Seemann 2018) from the obtained genome sequences of the ice plant. As comparative objectives, 25 kinds of 18S ribosomal genes from general crops (Japanese radish [ *Raphanus sativus*], Soybean [ *Glycine max*], Japanese trefoil [ *Lotus japonicus*], Barrelclover [ *Medicago truncatula*], Adzuki bean [ *Vigna angularis*], Banana [ *Musa acuminata*], Barley [ *Hordeum vulgare*], Sorghum [ *Sorghum bicolor*], Bread wheat [ *Triticum aestivum*], Maize [ *Zea mays*], Apple [ *Malus domestica*], Peach [ *Prunus persica*], Coffee tree (Arabica var.) [ *Coffea arabica*], Coffee tree (Robusta var.) [ *C. canephora*], Clementine [ *Citrus clementina*], Orange [ *C. sinensis*], Poplar, Tobacco [ *Nicotiana tabacum*], Tomato [ *Solanum lycopersicum*], Eggplant [ *S. melongena*], Potato [ *S. tuberosum*] and Grape [ *Vitis vinifera*]) were selected using the SILVA database (Release. 2020-08; Pruesse *et al.* 2007). After joining all ribosomal DNA sequences into one file, a molecular phylogenetic tree was created using implemented in NGPhylogeny.fr ( Lemoine *et al.* 2019) (Released in 2019). SH-aLRT (Shimodaira-Hasegawa-approximate likelihood ratio test) ( Shimodaira and Hasegawa 1999) was used to determine the molecular phylogenetic tree.

### Detection of repetitive regions

Repetitive sequences were detected, and custom repeat libraries involving transposable elements and long terminal repeat-retro transposons were generated using RepeatModeler2 (v2.0.2; Flynn *et al.* 2020) and TEclass (v2.1.3; Abrusán *et al.* 2009). Known repeat sequences were detected and classified in the assembled genome sequence with reference to the Repbase library ( Bao *et al.* 2015) and the custom repeat libraries, using RepeatMasker (v4.1.2-p1; Smit *et al.* 2013-2015). The capital letters in the genome sequences were replaced with small characters as soft masking.

### Search for genomic sequences coding transfer RNA (tRNA) and micro-RNA (miRNA)

The tRNA genes were identified in the draft common ice plant genome using tRNAscan-SE2.0 (v2.0.9) ( Chan *et al.* 2021). The tRNA data of other nine plant species— *Arabidopsis*, rice, tomato, poplar, horseradish, potato, grape, soybean, and coffee tree (robusta species)—were obtained from the PlantRNA database ( Cognat *et al.* 2013). The percentages of arbitrary tRNAs against the total tRNAs in the genome were calculated and compared to the ice plants’ values with those of the other species. Smirnov-Grubbs’ outlier tests were performed to select tRNAs more significantly involved. The test statistic T was calculated using the following equation:

$$T=\frac{\left(\text{Percentage of arbitrary tRNAs in the}\;\mathrm{ice}\;\text{plant}\right)-\left(\text{Sample mean for}\;\mathrm{all}\;\text{nine species}\right)}{\sqrt{\text{Sample variance}}}$$

The miRNA loci in the genome sequence were identified using the cmscan command in Infernal (v1.1.4; Nawrocki and Eddy 2013) using Rfam.

### Gene prediction

The BRAKER2 pipeline (v2.1.5; Brůna *et al.* 2021) was used for the prediction of genes in the common ice plant genome. Amino acid sequences were translated from the transcriptome profile reported by Lim *et al.* (2019) and used as additional reference data for the prediction of genes. BRAKER2 was used with the default parameters (–softmasking). The total sequences, total bases, total amino acids, and N50 were computed based on the resulting fasta-format files containing information about the genes, coding sequences, and amino acids using seqkit (v2.0.0; Shen *et al.* 2016) [key parameter: seqkit stats]. Protein BLAST searches ( *E*-value < 1e-5) were conducted using DIAMOND (v2.0.13.151; Buchfink *et al.* 2021) against the NCBI-non-redundant protein sequences (retrieved from NCBI in March 2022), Uniprot-swissprot (retrieved in March 18), Ensemble TAIR10 (retrieved in March 2022), and NCBI poplar amino acid sequence databases (retrieved from NCBI in March 2022).

### Protein domain searches

The protein domains in the genome were identified using the Pfam (v33.1) database ( Mistry *et al.* 2021) with *E*-value < 1e-3, using HMMER (v3.1b2; Potter *et al.* 2018). The protein databases of rice, maize, and poplar from the NCBI (last accessed February 2022) were used in the domain for a detailed classification of the PKinase family, the iTAK (v18.12) web tool ( Zheng *et al.* 2016; last accessed February 2022) was utilized. The ratio of families with a high ratio of genes to total genes in the ice plant was compared with that of the same families in the other plants. For statistical analysis, we used Smirnov-Grubbs’ outlier tests. The following equation was used to obtain the test statistic T:

$$T=\frac{\left(\text{Percentage of arbitrary protein families of the}\;\mathrm{ice}\;\text{plants}\right)-\left(\text{Sample mean for}\;\mathrm{all}\;\text{three species}\right)}{\sqrt{\text{sample variance}}}$$

Finally, BLASTP was used to compare proteins generated from the ice plant genome and those from *Arabidopsis*, rice, maize, and poplar and renamed TAIR10 ID. These IDs were subjected to gene ontology (GO) enrichment analysis using DAVID (updated in 2022; accessed on March 24; Sherman *et al.* 2022) based on a modified Fisher exact probability test with *E*-value < 0.05.

## Results

### Genome sequencing and *de novo* genome assembly

Short insert reads data (300 bp; Figure S1-(A), Sato *et al.* 2023b) with an estimated coverage of 50.92× and a ratio of unique to duplicate reads of about 1.63:1 was obtained by removing erroneous reads of raw paired-end data from the Illumina platform (BioProject: PRJDB13817; BioSample: SAMD00508673) (Table S1). The *M. crystallinum* genome size was estimated to be 366 to 369 Mb, with very low heterozygosity (about 0.010%) following an analysis of the frequency of 21 and 25-mers, using GenomeScope2.0 (Figure S1-(B) and (C), Sato *et al*. 2023b). The *M. crystallinum* final draft assembly included 286 Mb in 49,782 scaffolds with a scaffold N50 of 10,562 bp (Table S2). The BUSCO tool revealed 1,509 (93.49%) of 1,614 embryophyte library core genes, with 1,223 (75.77%) of these being ‘Complete’ matches in the genome. The completeness and contiguity of the genome were greater than the shotgun assembled *M. australis* and *S. chaucha* genomes (Figure S2: Sato *et al.* 2023b). Around 24,081 (99.5%) of the 24,204 transcripts from the transcriptome assembly of *M. crystallinum* leaves ( Lim *et al.* 2019), and all 135 DNA sequences registered in the NCBI, were aligned to the assembled genome (Supplementary Dataset S1: Sato *et al.* 2023c).

### Phylogenetic tree based on 18S ribosomal DNA sequences from 26 kinds of plants

We performed a phylogenic analysis using 18S ribosomal DNA (rDNA) among related species. The seven types of 18S rDNA were chosen from the ice plant genome sequence. Also, the other 25 plant species’ 18S rDNA sequences (see Phylogenetic tree creation among multiple plant species using 18S ribosomal DNA sequences in Methods) were retrieved from the ribosomal RNA database in SILVA and were aggregated with the ice plant 18S rDNAs. Based on these sequences, a molecular phylogenetic tree of 18S rDNA was constructed using PhyML+SMS/One Click in NGPhylogeny.fr (Figure S3, Sato *et al.* 2023b). The results showed that five species of 18S rDNAs were relatively closely related to poplar’s 18S rDNA.

### Search and classification of repetitive regions

In the 286.0 Mb *M. crystallinum* genome, 2,423 distinct repetitive sequences families, accounting for 110.9 Mb (38.8%) of the genome, were identified using custom repeat libraries and Repbase ( Bao *et al.* 2015). This ratio was smaller than Shen *et al.*’s (2022) reported value (48.04%). In decreasing order of frequency, the annotated repetitive elements were unclassified 78.0 Mb (27.27%), retroelements 21.9 Mb (7.64%), long interspersed nuclear elements (LINE) 12.5 Mb (4.37%), long terminal repeats (LTR) 9.35 Mb (3.27%), and simple repeats 7.26 Mb (2.54%). Some retroelements were classified into subfamilies, including L1/CIN4 12.4 Mb (4.34%) and RTE/Bov-B 0.85 Mb (0.03%) in the LINE, and Ty1/Copia 4.90 Mb (1.71%) and Gypsy/DIRS1 4.35 Mb (1.52%) in the LTR ( Table 1).

**Table 1.** Classification results of repetitive sequences in the ice plant genome.

Group	Number of elements	Length occupied, [bp]	Percentage of sequence, [%]
Retroelements ^(1)^	42,672	21,857,207	7.64
LINEs ^(2)^:	25,415	12,509,921	4.37
RTE/Bov-B	278	84,896	0.03
L1/CIN4	25,137	12,425,025	4.34
LTR elements ^(3)^:	17,257	9,347,286	3.27
Ty1/Copia	9,514	4,904,145	1.71
Gypsy/DIRS1	7,528	4,345,997	1.52
DNA transposons ^(4)^	5,725	2,789,199	0.98
hobo-Activator	728	285,258	0.10
Tc1-IS630-Pogo	237	160,874	0.06
Tourist/Harbinger	590	263,259	0.09
Rolling circles	121	112,702	0.04
Unclassified:	392,582	77,986,137	27.27
Total interspersed repeats:		102,632,543	35.88
Simple repeats:	137,610	7,255,343	2.54
Low complexity:	19,059	911,926	0.32

^(1)^ Retroelements: DNA sequences derived from viruses.

^(2)^ LINEs: Long interspersed nuclear elements.

^(3)^ LTR elements: Retrotransposons with long terminal repeat.

^(4)^ DNA transposons: DNA sequences moving through the genome.

### Detection of tRNA and miRNA coding genes from the genome

**Figure 1.**
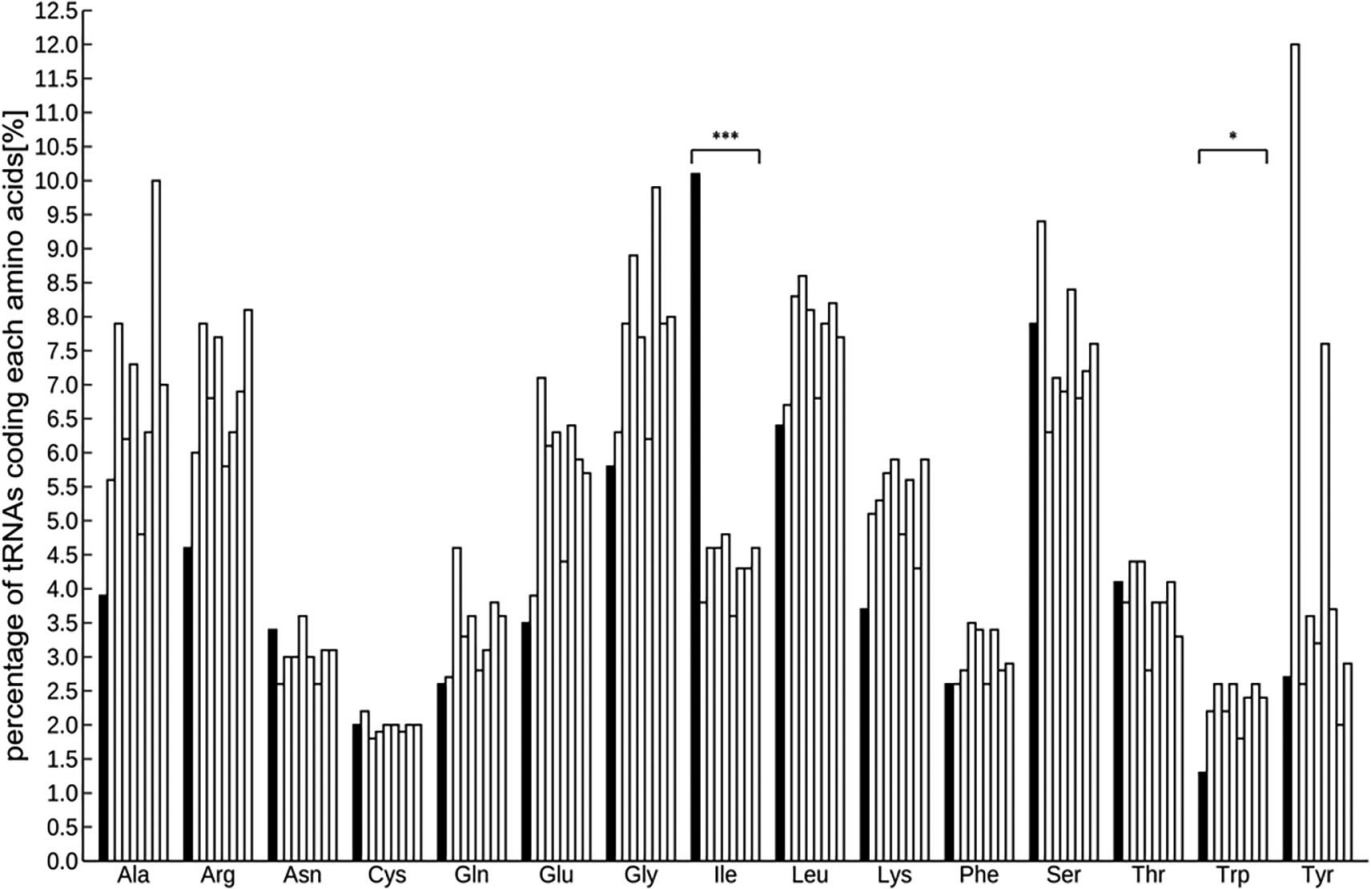
Comparison of the percentage of tRNAs in 9 plant species including ice plant. tRNAs significant differently abundant from the other 8 species by Smirnov-Grabs outlier test, are shown in black, and the other tRNAs are shown in gray. Bars indicate ice plant, *Arabidopsis*, rice, tomato, poplar, horseradish, potato, grape, and soybean from the left of each series. Asterisks indicate statistical significance: * *P* < 0.05, n = 9.

A total of 971 tRNAs, excluding pseudogenes, were detected in the assembled genome, and were sorted into several groups based on codon designation. The codon with the most abundant tRNA was isoleucine and the least was tryptophan (Figure S4, Sato *et al*. 2023b). The number of tRNAs was as follows: *Arabidopsis* 585, rice 505, poplar 505, tomato 723, horseradish 500, potato 736, grape 391, and soybean 700. Interspecific comparisons using the Smirnov-Grubbs outlier test and focusing on these eight species indicated that the abundance of isoleucine was significantly highest and that of tryptophan was significantly lower ( *P* < 0.05; Figure 1 and Table S3, Sato *et al*. 2023b).

In addition, miRNAs loci were identified from the genome with reference to the Rfam database, to obtain miRNA profiling independent of their expression levels. MiRNAs are 21 to 24 nt molecules that regulate post-transcriptional mRNA modification, playing important roles in plant growth and tolerance to environmental stress. 100 miRNA loci were identified and categorized into 25 families. The RNA family with the largest number of loci was MIR169 (25), followed by mir-399 (16), MIR159 (8), and mir-166 (7). mRNAs targeted by miRNA families were predicted (Table S4). For instance, MIR169 family miRNAs were presumed to bind to mRNAs encoding nuclear factor gamma subunit A (NF-YA) (Chiang *et al.* 2016). Overall, 13 types of 25 miRNA families were likely to target mRNAs encoding transcription factors: MYB33, MYB65, HD-ZIP, WRKY, AP2-like, NAC, ARFs, IAR3, ARF16, OsSPL14, SPL, GRF2, and HLH. The rest of the targeted mRNAs are anticipated to have functions in processes such as miRNA maturation, mRNA cleavage, or metal binding.

### Gene prediction and annotation

Genes (34,223), coding sequences (35,702), and amino acid regions (35,702) were predicted from the soft-masked draft *M. crystallinum* scaffolds *ab initio* using a homology-based pipeline in BRAKER2 using transcriptome data (Table S4). The representative value on bases showed that coding sequence regions cover at least 10.6% (30.4 Mb) of the total genome sequence. In comparison to several databases on 25 plant species’ genes registered in PGDBj ( Asamizu *et al.* 2014; last accessed in March 2022), the ice plants’ genes were as abundant as those of *Sorghum bicolor* and *Arabidopsis lyrate.* Additionally, summarized data indicated that the *M. crystallinum* gene number was 16 times larger than those of *S. bicolor* and *A. lyrate*, equivalent to about 27.6% of the number of genes of *Triticum aestivum* (bread wheat) and 3.31-fold greater than that of *Pyropia yezoensis* (bangia) (Figure S5, Sato *et al.* 2023b). Each translated protein sequence was used in a BLASTP search with the DIAMOND program ( Buchfink *et al.* 2021) against four kinds of protein sequence databases. In order of the proportion of homologous amino acid sequences identified, they were NCBI-non-redundant (82.59%), poplar (70.65%), TAIR10 (65.39%), and Swiss-prot (56.05%; Table 2) (Supplementary Dataset S2: Sato *et al.* 2023d). To simplify gene ID conversion to GO terms, the results, including TAIR ID, were used in the functional estimation.

**Table 2.** Statistics on coding sequences (CDS) and amino acid sequences predicted from the ice plant genome.

Regions	Whole genome	Genes	CDS	Amino acids
Sequences	49,782	34,223	35,702	35,702
Total bases, [nt] or amino acids, [aa]	286,008,514	91,283,320	30,364,506	9,958,616
Average length, [nt/aa]	5,745.20	2,667.30	1,185	411.1
Median length, [nt/aa]	3,357	1,761	950	330
N50, [nt/aa]	10,681	4,346	1,511	509

### Functional estimation and comparison of genomes

**Figure 2.**
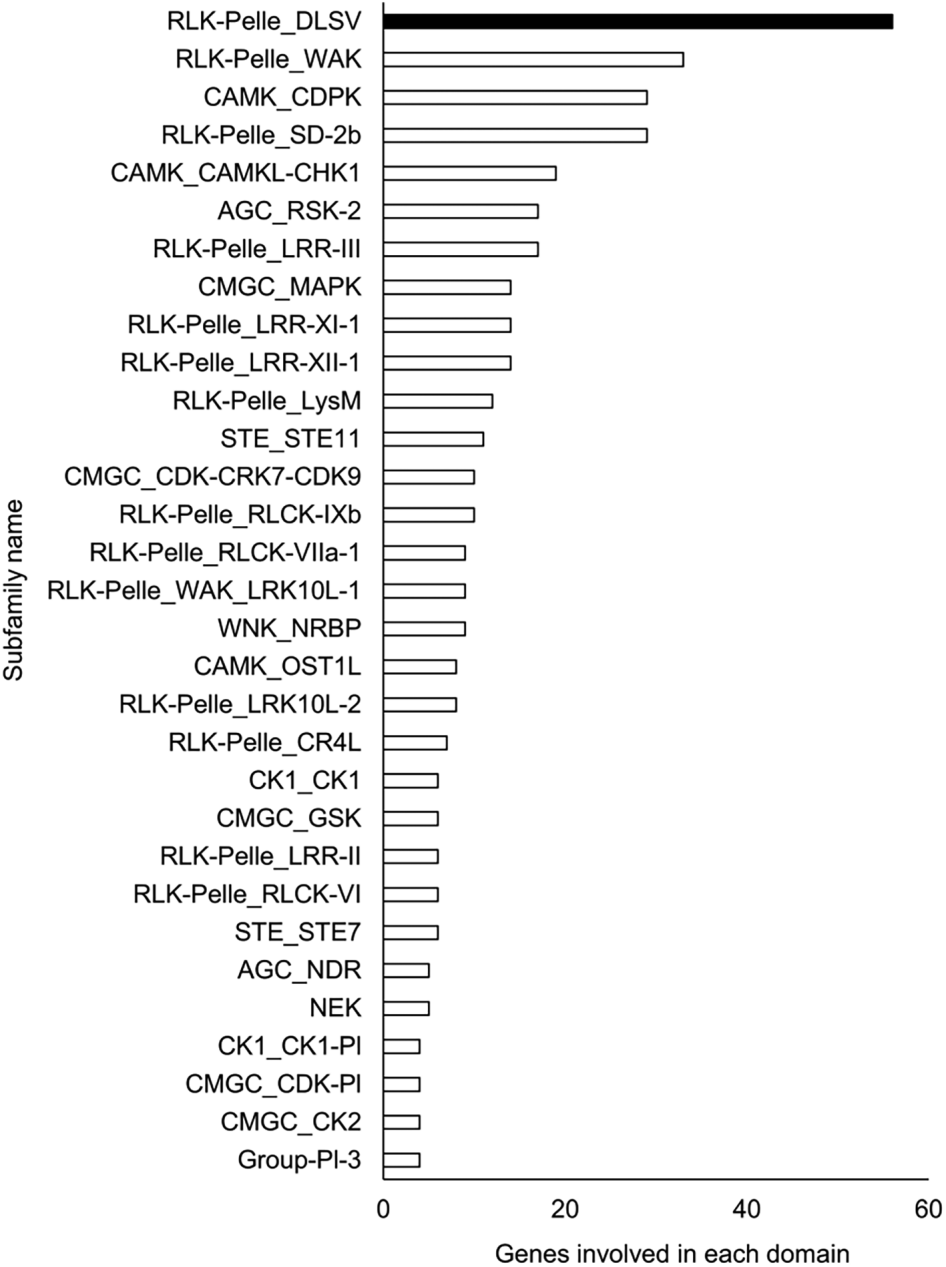
Top 30 Pkinase subfamilies classified in descending order of the number of genes included in them. The family with the highest number of genes is shown in black, and the other families are shown in white.

**Figure 3.**
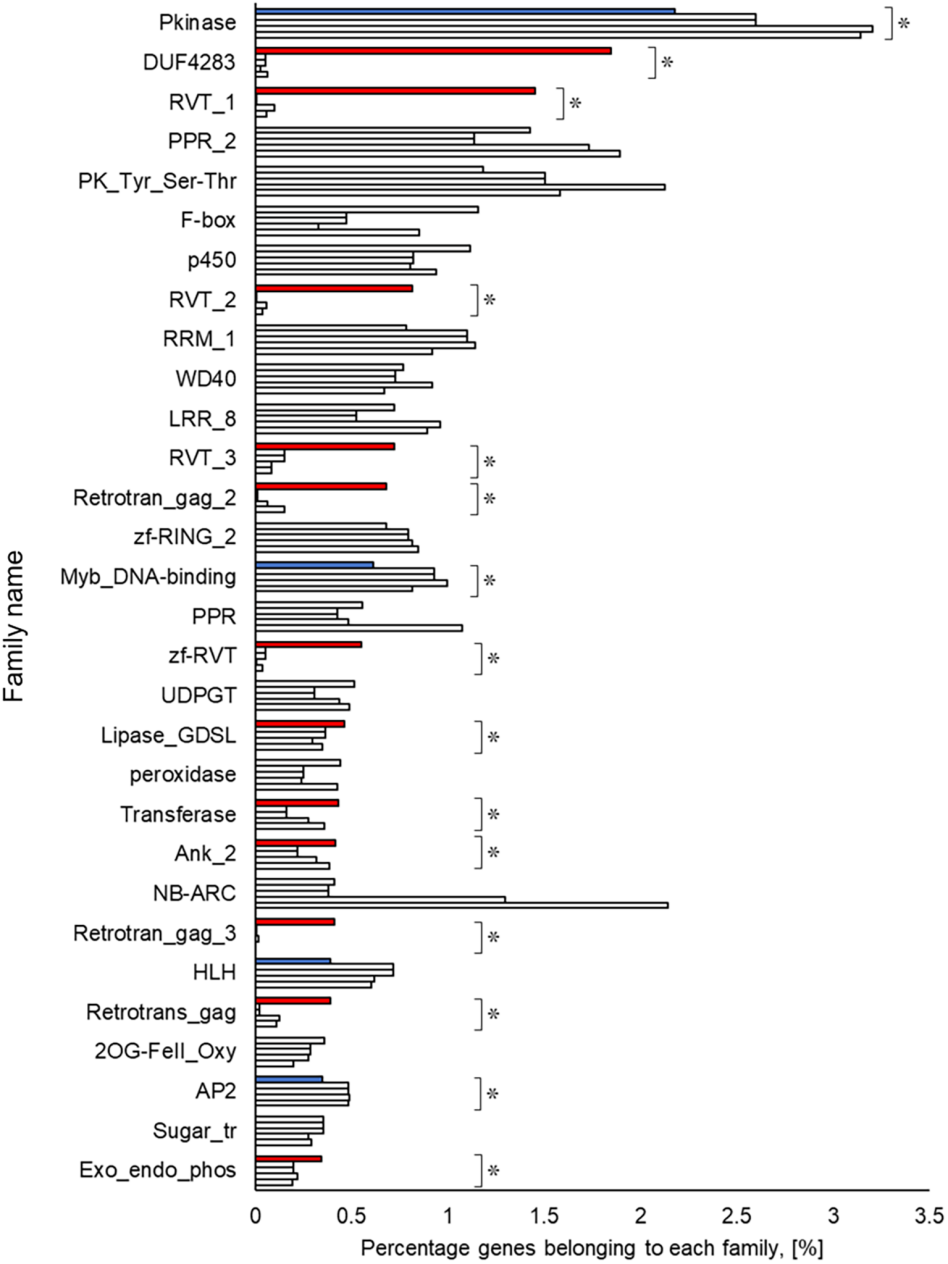
Top 30 gene families obtained from amino acid sequences detected in ice plant four other plant species. The top row for each family shows ice plant, *Arabidopsis*, rice, maize, and poplar. The independence of the proportion of genes belonging to a family in the ice plant is displayed using the Smirnov-Grubbs rejection test. Asterisks (*) indicate statistical significance: *P* < 0.05, n = 5. Independence is shown in red if the proportion is independently high in ice plant, in blue if it is low, and in gray if there is no difference.

A Pfam domain search based on the Pfam ( Mistry *et al.* 2021) database identified 3,703 domains in 23,521 (97.1%) genes. The most frequently occurring domain was the protein kinase domain (PKinase), at 2.18%, followed by a domain of unknown function (DUF) 4238 (1.85%), reverse transcriptase (RVT)_1 (1.45%), PPR domain-containing protein (PPR)_2 (1.42%), and protein tyrosine and serine/threonine kinase (PK_Tyr_Ser-Thr) (1.18%) (Supplementary Dataset S3: Sato *et al.* 2023e). The PKinase family was further classified into 94 kinase families using iTAK ( Zheng *et al.* 2016). The top 30 kinase families with the largest number of ice plant genes are shown in descending order in Figure 2. Compared to the other four plant species, the proportion of 12 families was significantly higher ( *P* < 0.05), and eight families—DUF4238, RVT_1, RVT_2, RVT_3, Retrotrans_gag_2, zf_RVT, Retrotrans_gag_3, Retrotrans_gag—contained retroelement domains that could be attributed to a retrotransposable element ( Figure 3). The annotated genes were assigned to GO classifications based on TAIR ID in three groups —biological process (BP), cellular component (CC), and molecular function (MF)—and were categorized into 403 GO terms using the gene functional classification tool in the DAVID web service. The proportion of genes assigned to 94 GO terms did not differ significantly among five plant species ( *P* > 0.05; Figure S6 to S8, Sato *et al*. 2023b), indicating that they are essential to plant survival. These findings confirmed that the ice plant genome constructed in this study contained conserved genes to some extent. 18 GO terms were identified only from ice plants, although the number of genes was small (Table S6), involving virus resistance, pollen tube development, and fat biosynthesis (BP); cytoplasmic vesicle and “soluble NSF attachment protein receptor” (SNARE; CC); and *O*-acyltransferase for transferring fatty acids (MF).

## Discussion

*M. crystallinum* is utilized as a model plant for investigating halophilism, salt tolerance, and CAM photosynthesis. In this research, we have assembled the common ice plant’s genome sequence and elucidated the genome’s function in detail for the first time in this species. This genomic resource covers all protein-coding, non-transcribed, and untranslated regions. Our results of genomic functional analysis on *M. crystallinum* provide new insights into the molecular mechanisms underlying the plant’s adaptation to NaCl stress including conversion of the photosynthesis.

The total assembled genome length (286 Mb) was approximately 26% smaller than the genome size estimated using the experimental or bioinformatic method reported by Meyer *et al.* (1990; 390 Mb, de Rocher *et al.* (1990; 390 Mb), and Shen *et al.* (2022; 378 Mb). The genome size estimated using k-mer distribution analysis is likely to be smaller than that using experimental data, including flow cytometry, given the effects of repetitive sequences and other obscure nucleotide sequences ( Bennett *et al.* 2003; Al-Qurainy *et al.* 2021). Barkla *et al.* (2018) have shown that the ploidy levels of the leaf increased throughout its development. Because polyploidy is becoming a concern when NGS is used for genome assembly ( Kyriakidou *et al.* 2018), the endopolyploidy of ice plant leaves may increase the complexity of genome assembly. Experimental data is supposed to help to support the present results and determine the exact ice plant genome size.

Interestingly, the phylogenic tree analysis indicated that the genome composition of the ice plant was similar to that of the poplar. Previous studies have reported poplar-derived genes for salt tolerance, including PtNF-YA9 ( Lian *et al.* 2018), PtSAP13 ( Li *et al.* 2019), and PtVP1.1 ( Yang *et al.* 2015). These results suggest that the ice plant genome constructed in this study is a highly conserved sequence that can be used for phylogenetic relationship analysis.

We found that the repetitive *M. crystallinum* sequences occupy 110.9 Mb (38.8%) of the genome. Advances in genomics over several decades have revealed that repetitive sequences play essential roles in regulating gene expression in higher plants. Recent studies observed that the transposable elements, involving many repetitive sequences were highly expressed under heat, salt, and intense light stresses, in Arabidopsis, tomato, and mangrove species ( Deneweth *et al.* 2022; Wang *et al.* 2022). These results suggested that it affected the expression levels of nearby genes for transcriptional factors, including DREB, NAC, MYB, AP2/ERF, NF-Y, and Abscisic acid 8′-hydroxylase. Further studies indicated that cis-regulatory motifs associated with C4 photosynthesis, rate-determined by the same enzyme up to at least 669, and the non-coding RNAs regulating methyltransferases expression levels are derived from transposable elements ( Nosaka *et al.* 2012; Cao *et al.* 2016). Transposable element expression is suppressed by cytosine methylation in DNA sequences, chromatin remodeling, and degradation by small interfering RNA (siRNA; Ito 2013). These previous results suggested that the common ice plant has repetitive sequences with similar effects on gene expression regulation.

Two kinds of representative small non-coding RNAs were found in the ice plant genome—971 tRNAs and 100 miRNA loci—which are anticipated to be relevant to metabolic pathway and post-transcriptional modification. Generally, a tRNA recruits an amino acid corresponding to its codon, which means that the abundance of a specific tRNA is proportional to that of the relevant amino acid. Some studies have shown the effectiveness of amino acids in metabolism for environmental stress reduction. For example, 5-aminolevulinic acid, a key precursor in porphyrins biosynthesis, including chlorophyll and heme, can alleviate abiotic stresses, including salinity, drought, heat, cold, and UV-B ( Tan *et al.* 2022). The Smirnov-Grabs outlier test revealed that the isoleucine-specific tRNA was present at a significantly higher proportion in the ice plant’s genome than in eight other plant species. It is the precursor of JA-Ile, the active molecule of the plant hormone jasmonic acid, which has been implicated in pathogen resistance in plants ( Li *et al.* 2021). The least abundant coded tRNA was tryptophan, which serves as the melatonin precursor, a signaling molecule that regulates responses to abiotic stress, such as water shortage ( Sadak and Ramadan 2021). These results suggest that the abundance of amino acids in the ice plant may differ from those in the other eight plants, indicating the possible presence of different stress tolerance mechanisms.

Some miRNAs identified in the ice plant’s genome appeared to be key small molecules in the stability of mRNAs coding for epigenetic and transcription-related factors. NF-YA were targeted by 31 MIR169 loci known to integrally regulate gene expression by maintaining histone acetylation in soybeans ( Lu *et al.* 2021), or binding to circadian rhythm-related elements, including the “CCAAT” motif in *Arabidopsis* ( Wenkel *et al.* 2006; Zhao *et al.* 2016). Several miRNA-targeting transcription factors were associated with salt tolerance ( *HLH*, *SPL*, *HD-ZIP*) ( Shen *et al.* 2019; Wang *et al.* 2019, 2021) or CAM photosynthesis ( *WRKY*, *AP2*, *MYB*, *NAC*) ( Amin *et al.* 2019; Yuan *et al.* 2020; Shah *et al.* 2021). All target gene families were found in the protein family collection in the ice plant genome, except for *SPL* and *lectin receptor kinase* (see Supplementary Dataset S1, Sato *et al.* 2023c), indicating that an antagonistic relationship between miRNAs and mRNAs underlies the stress tolerance and photosynthetic conversion mechanisms of the ice plants. Additional miRNA sequence information is expected to provide more accurate data and form the basis for testing these assumptions.

The richest PKinase subfamily was “receptor-like kinase/Pelle, DUF26, SD-1, LRR-VIII and VWA, a moss-specific new RLK subfamily (RLK-Pelle_DLSV)”, containing primarily receptor-type kinases, which was consistent with the transcriptome profiling in a halophyte, *Nitraria sibrica* ( Zhang *et al.* 2022). It has been assumed to be involved in cell wall biosynthesis, adhesion, and developmental regulation. For instance, WAK, the second most frequent PKinase in the ice plant genome, has been reported to control cell wall expansion, metal resistance, and pathogen resistance ( Gish and Clark 2011). The common ice plants show halophilism or salt tolerance; a detailed study may help to shed light on the mechanism of this tolerance from the perspective of phosphorylation. In contrast to the rare PKinase, the richness of retrotransposon-derived domains (reverse transcriptase and gag genes), involved in RNA packaging and the replication cycle ( Orozco-Arias *et al.* 2019), was apparent in the ice plant compared to the other plant species. A recent study suggested a human retrotransposon-derived imprinted gene, paternally expressed gene 10 (PEG10), mediates cellular proliferation and inhibits apoptosis ( Golda *et al.* 2020). However, it remains unclear what effects these proteins have on the plants’ physiology. Our latest experiment demonstrated that the ice plant’s cell cycle-related genes were upregulated in the presence of 100 mM NaCl ( Sato *et al.* 2022), possibly implying an impact of retrotransposon-derived proteins on the cell division of the ice plant’s cells. Lipases, transferases, and phosphatases were abundant, and transcription factors such as Myb, HLH, and AP2 were scarce in the genome of the common ice plant. Two reviews show these enzymes and transcription factors assume a key role in plants’ survival under salinity ( Reyes-Pérez *et al.* 2019; Chaudhry *et al.* 2021), but it is not yet clear whether they interact with each other. Elucidation of these protein interactions by transcriptome and interactome analysis may provide crucial evidence about their unknown functions.

Finally, comparing the gene functions among the genomes of five plant species —ice plant, Arabidopsis, rice, maize, and poplar—based on their gene counts, 18 gene functions were found only in the ice plant. Previous studies (12 reviews and 11 research articles) with sophisticated experimental backgrounds indicated that all gene functions were possibly associated with the mechanisms of halophilism, salt tolerance, and photosynthetic conversion. These gene functions were categorized as related to biological defense, growth, reproduction, transcription, post-transcription, and intermembrane transportation. Therefore, focusing on the homologous of the ice plant genes with these functions may provide critical insight into the salt-induced growth and photosynthetic systems.

## Conclusion

We succeeded in assembling the M. crystallinum genome using Illumina PE reads, characterizing the genome, and identifying the potential gene, non-transcriptional and translational regions, and repetitive sequences. Furthermore, we made the ice plant genome available to all, which means the end of this plant’s genome information opacity temporarily. Our results revealed that salt tolerance increases with growth, and C3-CAM photosynthetic conversion in the presence of NaCl is probably controlled by both protein-coding genes and potential genomic factors, including transposable elements, tRNAs, miRNAs, and protein kinases. These findings provide new insights into the mechanisms of plant growth under environmental stresses and can be used to develop highly high salt-tolerant crops. We hope this study will be a good step stone to the developed genomic science of the common ice plant.

## Data Availability

DDBJ BioProject: *Mesembryanthemum crystallinum* genome assembly and analysis. Accession number PRJDB13817, https://ddbj.nig.ac.jp/resource/bioproject/PRJDB13817. DDBJ BioSample: *Mesembryanthemum crystallinum* metadata. Accession number SAMD00508673, https://ddbj.nig.ac.jp/resource/biosample/SAMD00508673. DDBJ Sequence Read Archive (DRA): Raw data from Illumina Novaseq 6000. Accession numbers DRA015289 and DRR424237, https://ddbj.nig.ac.jp/resource/sra-submission/DRA015289 and https://ddbj.nig.ac.jp/resource/sra-run/DRR424237. The assembled genome sequence and annotation information generated in this study are available at DDBJ ( http://getentry.ddbj.nig.ac.jp/top-j.html), accession number BSSO01000001-BSSO01049782. Protocol.io: Methods in “The first released available genome of the common ice plant ( *Mesembryanthemum crystallinum* L.) extended the research region on salt tolerance, C3-CAM photosynthetic conversion, and halophism” V.1. https://dx.doi.org/10.17504/protocols.io.6qpvr4qdogmk/v1 figshare: Supplementary_Information.pdf. https://doi.org/10.6084/m9.figshare.21788624 ( Sato *et al*., 2023b) figshare: Supplementary_Dataset_S1.xlsx. https://doi.org/10.6084/m9.figshare.21788666 ( Sato *et al*., 2023c) figshare: Supplementary_Dataset_S2.xlsx. https://doi.org/10.6084/m9.figshare.21788675 ( Sato *et al*., 2023d) figshare: Supplementary_Dataset_S3.xlsx. https://doi.org/10.6084/m9.figshare.21788681 ( Sato *et al*., 2023e) Data are available under the terms of the Creative Commons Attribution 4.0 International license (CC-BY 4.0).
