# Supplementary material for: The first released available genome of the common ice plant ( Mesembryanthemum crystallinum L.) extended the research region on salt tolerance, C 3-CAM photosynthetic conversion, and halophilism
Source: F1000Res. 2024 Jan 4;12:448. Originally published 2023 Apr 27. [Version 4] doi: 10.12688/f1000research.129958.4 (PMC11016173; doi:10.12688/f1000research.129958.4)
Supplement: Peer review history

# Reviewer report: Reviewer response for version 4

**DOI:** 10.5256/f1000research.160684.r261672

**Published:** 2024-04-23

**Authors:** Zbigniew Miszalski

**Affiliations:** 1 Jagiellonian University, Kraków, Poland

Comments to MS „ **The first released available genome of the common ice plant ( *Mesembryanthemum crystallinum* L.) extended the research region on salt tolerance, C _3_-CAM photosynthetic conversion, and halophilism**”  

 In ‘80 it was demonstrated many times that salinity play an important role in aclimatory and defense mechanisms in *Mesembryanthemum crystallinum* L., however, our knowledge about specific gene induction during NaCl application to the soil is rather scant. The analysis of the effects caused by salinity stress on physiology of glycophytes with focus on reactive species and antioxidative machinery occurring in different plant organs was described in many other original and review papers. It was also demonstrated many times that different stress factors and resulting level of reactive oxygen species play an important regulatory role in acclimatory and defense mechanisms in all plants (glycophytes and halophytic). There have been few studies up to now of the biochemical consequences salinity on semi-halophytic plants, which, may be expected to be particularly important during next decades. The problem is well stated however it needs some additional information explaining why this model plant is that important. There are many novel aspects of this work that extend beyond what has previously been published in the literature. The general intention is excellent. It is really some very fine experimental work helping to solve many questions.

 This manuscript focuses on changes in some genetic mechanisms in plants exposed to stress. The data presented here are interesting and they need some additional information and adequate interpretation. There are some issues the authors should address before this manuscript could be considered acceptable.

 Major criticism:

 It is well known that changes in photosynthetic machinery (C3-CAM) are related with big changes in metabolism and this can be discussed in terms of stress signal. We know that during C3-CAM shift daily **metabolic-photosynthetic rhythm** is totally different (activity and gene induction). Interestingly photosynthetic changes are **reversible**, and this is also under genetic control. Thus, results shown in this MS can be used also to analyze related questions. We know also that this plant was used as model plant in research focusing on induction of antioxidative mechanisms. We know that some ROSs produced in the presence NaCl stressor play a role as a signal molecules and, with cross tolerance mechanism, can adapt plants to other stresses. It was described that this plant is very resistant to **some heavy metals**. It was also described that C3 and CAM performing plant differ in their resistance to **biotic stresses** (bacterial and fungi). This would help to understand plant reaction in given circumstances. It would be interesting to mention this is edible plant. **All these aspects and these results are not even mentioned in this MS.**

**Discussion**

 All changes mentioned in Discussion can be important when analyzing different stresses and not only salinity. Different ROS compounds show differentiated reactivity with biomolecules and play differentiated role in complex **signalling system**. Plants developed several systems to control the level of ROS compounds, but on the other side, they produce them. Plants need ROSs and stress according to the rule “no stress no development”. Thus, high activity of antioxidant machinery can be responsible for sensitivity/tolerance to many stresses. **Such possibility should be indicated in this MS.**

 Discussion should also point out the novel aspects of presented results for physiology of **halophytic and semi-halophytic** plants. This would help to understand what is plant reaction to stress. The meaning of presented results are discussed in terms of possible changes in plants exposed to stresses, but the discrepancies in changes of all enzymes are not discussed.

 From the presented MS we learn  number of  details, however, our knowledge about some facts often is presented as totally negative, and its regulative role is often forgotten.

 It was shown that **cis-regulatory motifs are associated with C4 photosynthesis derived from transposable elements**. It may be expected that this is strongly connected with **CAM induction**. Similarly the function of small non-coding RNAs and regulation of posttranscriptional modification can be crucial.

Is the work clearly and accurately presented and does it cite the current literature?

Partly

If applicable, is the statistical analysis and its interpretation appropriate?

Yes

Are all the source data underlying the results available to ensure full reproducibility?

Yes

Is the study design appropriate and is the work technically sound?

Yes

Are the conclusions drawn adequately supported by the results?

Partly

Are sufficient details of methods and analysis provided to allow replication by others?

Yes

Reviewer Expertise:

plant physiology

I confirm that I have read this submission and believe that I have an appropriate level of expertise to confirm that it is of an acceptable scientific standard, however I have significant reservations, as outlined above.

# Reviewer report: Reviewer response for version 4

**DOI:** 10.5256/f1000research.160684.r235520

**Published:** 2024-04-13

**Authors:** Xin-Guang Zhu

**Affiliations:** 1 Institute of Plant Physiology and Ecology, Chinese Academy of Sciences, Shanghai, 200032, China

I endorse the indexing of this manuscript. The revision effectively addressed my earlier concerns.

Is the work clearly and accurately presented and does it cite the current literature?

Yes

If applicable, is the statistical analysis and its interpretation appropriate?

I cannot comment. A qualified statistician is required.

Are all the source data underlying the results available to ensure full reproducibility?

No source data required

Is the study design appropriate and is the work technically sound?

Partly

Are the conclusions drawn adequately supported by the results?

Partly

Are sufficient details of methods and analysis provided to allow replication by others?

Yes

Reviewer Expertise:

NA

I confirm that I have read this submission and believe that I have an appropriate level of expertise to confirm that it is of an acceptable scientific standard.

# Reviewer report: Reviewer response for version 3

**DOI:** 10.5256/f1000research.153065.r215671

**Published:** 2023-11-30

**Authors:** Xin-Guang Zhu

**Affiliations:** 1 Institute of Plant Physiology and Ecology, Chinese Academy of Sciences, Shanghai, 200032, China

The study reports the sequencing of the genome of common ice plant, reports the detailed genome structure, genes, lncRNAs, transposons etc in the genome. The information provided in this study will certainly benefit the future study on ice plant.

 However, since this study did not include detailed physiological studies or transcriptomics studies, etc., the conclusions drawn on the potential role of either genes or lncRNA or other gene components on plant development, either the CAM development etc., are not well grounded. It is completely appropriate to describe the genome information and the genes etc in ice plant. However, to assign particular function to a gene or a group of genes, detailed experiments need to be conducted.

 Therefore, on one hand, I found that the genome information provided from this study is highly valuable; on the other hand, the claims or statements on the functions of the genes in the genome are not grounded. So, I think this paper needs to be substantially revised to be acceptable for indexing.

Is the work clearly and accurately presented and does it cite the current literature?

Yes

If applicable, is the statistical analysis and its interpretation appropriate?

I cannot comment. A qualified statistician is required.

Are all the source data underlying the results available to ensure full reproducibility?

No source data required

Is the study design appropriate and is the work technically sound?

Partly

Are the conclusions drawn adequately supported by the results?

Partly

Are sufficient details of methods and analysis provided to allow replication by others?

Yes

Reviewer Expertise:

NA

I confirm that I have read this submission and believe that I have an appropriate level of expertise to state that I do not consider it to be of an acceptable scientific standard, for reasons outlined above.

# Response

**Published:** 2023-12-26

**Authors:** Ryoma Sato

**Affiliations:** Kyushu University, Fukuoka, Fukuoka, Japan

Dear Zhu,

 We appreciate the opportunity to submit an updated version of our manuscript, titled "The first released available genome of the common ice plant ( *Mesembryanthemum crystallinum *L.) extended the research region on salt tolerance, C _3_-CAM photosynthetic conversion, and halophilism," to *F1000 Research*. We are deeply grateful for the time you spent and insightful feedback to be instrumental in enhancing our paper. We are delighted to resubmit our work for your renewed evaluation. As the revisions throughout our paper align with the thoughtful recommendations you offered, we trust that the detailed responses provided below will adequately address all your concerns and points.

 To assist in your assessment of our revised manuscript, we present responses in detail to each of the queries and comments outlined in your message dated November 30 ^th^.

*>The study reports the sequencing of the genome of common ice plant, reports the detailed genome structure, genes, lncRNAs, transposons etc in the genome. The information provided in this study will certainly benefit the future study on ice plant.*

 Response: We appreciate your recognition of the significance of our work in providing the genome sequence of common ice plants. We believe it is a substantial contribution to the scientific community.

*>However, since this study did not include detailed physiological studies or transcriptomics studies, etc., the conclusions drawn on the potential role of either genes or lncRNA or other gene components on plant development, either the CAM development etc., are not well grounded.*

 Response: We have concurred with your suggestion and implemented it in our Discussion section, resulting in the removal or revision of eight instances of speculative content (pages 12 to 14), and the exclusion of six references from the list, (pages 15 to 22). However, we have retained some of our arguments because we believe that my discussions in this paper are based on scientific experiments, as they are all referenced from peer-reviewed papers.

 We have removed six sentences in the Discussion section as follows:

 ・For instance, WAK, the second most frequent PKinase in the ice plant genome, has been reported to control cell wall expansion, metal resistance, and pathogen resistance ( Gish and Clark 2011).

 ・For example, 5-aminolevulinic acid, a key precursor in porphyrins biosynthesis, including chlorophyll and heme, can alleviate abiotic stresses, including salinity, drought, heat, cold, and UV-B ( Tan *et al.* 2022).

 ・A recent study suggested a human retrotransposon-derived imprinted gene, paternally expressed gene 10 (PEG10), mediates cellular proliferation and inhibits apoptosis ( Golda *et al.* 2020). However, it remains unclear what effects these proteins have on the plants’ physiology.

 ・” ; a detailed study may help to shed light on the mechanism of this tolerance from the perspective of phosphorylation “ in “It has been assumed to be involved in cell wall biosynthesis, adhesion, and developmental regulation. The common ice plants show halophilism or salt tolerance; a detailed study may help to shed light on the mechanism of this tolerance from the perspective of phosphorylation.”

 ・Our latest experiment demonstrated that the ice plant’s cell cycle-related genes were upregulated in the presence of 100 mM NaCl ( Sato *et al.* 2022), possibly implying an impact of retrotransposon-derived proteins on the cell division of the ice plant’s cells.

 ・These results suggested that it affected the expression levels of nearby genes for transcriptional factors, including DREB, NAC, MYB, AP2/ERF, NF-Y, and Abscisic acid 8′-hydroxylase.

 Also, we have revised some groups of sentences in Discussion section and enhanced the change by bold as follows:

 Before revision:

 Our results of genomic functional analysis on *M. crystallinum* provide new **insights into **the molecular mechanisms underlying the plant’s adaptation to NaCl stress including conversion of the photosynthesis.

 After revision:

 Our results of genomic functional analysis on *M. crystallinum* provide **new clue to solve** the molecular mechanisms underlying the ice plant’s adaptation to NaCl stress including conversion of the photosynthesis.

 Before revision:

 The Smirnov-Grabs outlier test **revealed that the isoleucine-specific tRNA was present at a significantly higher proportion in the ice plant’s genome than in eight other plant species. It is the precursor of JA-Ile, the active molecule of the plant hormone jasmonic acid, which has been implicated in pathogen resistance in plants (** **Li *et al.* 2021** **). The least abundant coded tRNA was tryptophan, which serves as the melatonin precursor, a signaling molecule that regulates responses to abiotic stress, such as water shortage (** **Sadak and Ramadan 2021** **). These results suggest that the abundance of amino acids in the ice plant may differ from those in the other eight plants, indicating the possible presence of different **stress tolerance mechanisms.

 After revision:

 The Smirnov-Grabs outlier test **indicated a notable abundance of tRNAs coding for isoleucine and a scarcity for tryptophan, pointing to a unique amino acid distribution in the ice plant compared to eight other plants, potentially contributing to its **stress tolerance mechanisms.

 In addition, we excluded the following six references listed below:

 Gish LA, Clark SE: The RLK/Pelle family of kinases. *Plant J.* 2011;66(1):117–127. 21443627 10.1111/j.1365-313X.2011.04518.x PMC4657737

 Tan S, Cao J, Xia X, et al.: Advances in 5-aminolevulinic acid priming to enhance plant tolerance to abiotic stress. *Int. J. Mol. Sci.* 2022;23:702. 35054887 10.3390/ijms23020702 PMC8775977

 Golda M, Mótyán JA, Mahdi M, et al.: Functional study of the retrotransposon-derived human PEG10 protease. *Int. J. Mol. Sci.* 2020;21(7):2424. 32244497 10.3390/ijms21072424 PMC7212762

 Sato R, Tran DQ, Yoshida K, et al.: NaCl–promoted respiration and cell division in halophilism of a halophyte, the common ice plant *Mesembryanthemum crystallinum* L. *J. Fac. Agric. Kyushu Univ.* 2022;67(2):153–164. 10.5109/4797822

 Li Y, Li S, Du R, et al.: Isoleucine enhances plant resistance against Botrytis cinerea via jasmonate signaling pathway. *Front. Plant Sci.* 2021;12:628328. 34489985 10.3389/fpls.2021.628328 PMC8416682

 Sadak MS, Ramadan AAEM: Impact of melatonin and tryptophan on water stress tolerance in white lupine ( *Lupinus termis* L.). *Physiol. Mol. Biol. Plants.* 2021;27(3):469–481. 33854277 10.1007/s12298-021-00958-8 PMC7981349

 > *It is completely appropriate to describe the genome information and the genes etc in ice plant. However, to assign particular function to a gene or a group of genes, detailed experiments need to be conducted.*

 Response:

 Thank you for the positive evaluation of our work to provide the whole genome information as stated in 'It is completely appropriate to describe the genome information and the genes etc in ice plant.’ In addition, you have raised an important observation that 'to assign a particular function to a gene or a group of genes, detailed experiments need to be conducted'; however, we believe that detailed experiments would be outside the scope of our research paper because this research aims to register the genome sequence of the common ice plant to public databases to accelerate physiological research. We plan to publish the profiling of CAM-related genes based on transcriptome data as a section in the paper on the decoding of the ice plant's genome sequence using long reads in *Scientific Reports*. Therefore, we would like you to be expected for further experiments and follow-up reports.

*>Therefore, on one hand, I found that the genome information provided from this study is highly valuable;*

 We thank you again for your agreement with the importance of deciphering the genome sequence of the common ice plants and providing it to researchers worldwide. In connection, our decoding genome sequence became publicly accessible on May 5th, 2023, at the National Center for Biological Information (NCBI) in addition to the DNA Data Bank of Japan (DDBJ). Consequently, we have replaced the text in the 'Data availability' section under 'Underlying data' as follows:

 Before replacement:

 DDBJ BioProject: *Mesembryanthemum crystallinum* genome assembly and analysis. Accession number PRJDB13817, https://ddbj.nig.ac.jp/resource/bioproject/PRJDB13817.

 DDBJ BioSample: *Mesembryanthemum crystallinum* metadata. Accession number SAMD00508673, https://ddbj.nig.ac.jp/resource/biosample/SAMD00508673.

 DDBJ Sequence Read Archive (DRA): Raw data from Illumina Novaseq 6000. Accession numbers DRA015289 and DRR424237, https://ddbj.nig.ac.jp/resource/sra-submission/DRA015289 and https://ddbj.nig.ac.jp/resource/sra-run/DRR424237.

 The assembled genome sequence and annotation information generated in this study are available at DDBJ ( http://getentry.ddbj.nig.ac.jp/top-j.html), accession number BSSO01000001-BSSO01049782.

 After replacement:

 DDBJ and NCBI BioProject: *Mesembryanthemum crystallinum* genome assembly and analysis. Accession number PRJDB13817, https://ddbj.nig.ac.jp/resource/bioproject/PRJDB13817 and https://www.ncbi.nlm.nih.gov/bioproject/PRJDB13817/

 DDBJ and NCBI BioSample: WGS_iceplant. Accession number SAMD00508673, https://ddbj.nig.ac.jp/resource/biosample/SAMD00508673 and https://www.ncbi.nlm.nih.gov/biosample/SAMD00508673

 DDBJ Sequence Read Archive (DRA) and NCBI Sequence Read Archive (SRA): Raw data from Illumina Novaseq 6000. Accession numbers DRA015289, https://ddbj.nig.ac.jp/resource/sra-submission/DRA015289 and https://www.ncbi.nlm.nih.gov/sra/?term=DRA015289

 The assembled genome sequence and annotation information generated in this study are available at DDBJ ( http://getentry.ddbj.nig.ac.jp/top-j.html), accession number BSSO01000001-BSSO01049782, and NCBI (https://www.ncbi.nlm.nih.gov/datasets/genome/GCA_030267885.1/), accession number GCA_030267885.1.

 > *on the other hand, the claims or statements on the functions of the genes in the genome are not grounded. So, I think this paper needs to be substantially revised to be acceptable for indexing.*

 As mentioned above, this genome sequence is the first to be shared in at least two major biological databases, resulting in the resolution of the issue that 'The poor transparency of genome information causes the delayed elucidation of whole-genome functions of the common ice plants dominating not only photosynthetic conversion systems but also halophilism and salt tolerance' mentioned in the 'Introduction' of this paper. Therefore, we expect that this research article is published with indexing as soon as possible.

 Below are the minor changes made to the manuscript, other than those mentioned above.

 Before: Finally, comparing the gene functions among the genomes of five plant species —ice plant, Arabidopsis, rice, maize, and poplar—based on their gene counts, 18 gene functions were found only in the ice plant.

 After: Finally, comparing the gene functions among the genomes of five plant species —the common ice plant, Arabidopsis, rice, maize, and poplar—based on their gene counts, 18 gene functions were found only in the ice plant.

 Before: Interestingly, the phylogenic tree analysis suggested that the genome composition of the ice plant was similar to that of the poplar.

 After: Interestingly, the phylogenic tree analysis suggested that the genome composition of the ice plant was similar to that of the poplar.

 Before: NF-YA were targeted by 31 MIR169 loci known to integrally regulate gene expression by maintaining histone acetylation in soybeans ( Lu *et al.* 2021), or binding to circadian rhythm-related elements, including the “CCAAT” motif in *Arabidopsis* ( Wenkel *et al.* 2006; Zhao *et al.* 2016). Several miRNA-targeting transcription factors were associated with salt tolerance ( *HLH*, *SPL*, *HD-ZIP*) ( Shen *et al.* 2019; Wang *et al.* 2019, 2021) or CAM photosynthesis ( *WRKY*, *AP2*, *MYB*, *NAC*) ( Amin *et al.* 2019; Yuan *et al.* 2020; Shah *et al.* 2021).

 After: The mRNA *NF-YA* were targeted by 31 MIR169 loci known to integrally regulate gene expression by maintaining histone acetylation in soybeans ( Lu *et al.* 2021), or binding to circadian rhythm-related elements, including the “CCAAT” motif in *Arabidopsis* ( Wenkel *et al.* 2006; Zhao *et al.* 2016). Several miRNA-targeting transcription factors were associated with salt tolerance ( *HLH*, *SPL*, and *HD-ZIP*) ( Shen *et al.* 2019; Wang *et al.* 2019, 2021) or CAM photosynthesis ( *WRKY*, *AP2*, *MYB*, and *NAC*) ( Amin *et al.* 2019; Yuan *et al.* 2020; Shah *et al.* 2021).

 Thank you once more for the chance to enhance our manuscript with your insightful remarks and questions despite your busy schedule. We have diligently applied your suggestions and trust that these improvements will convince you to approve our submission.

# Reviewer report: Reviewer response for version 2

**DOI:** 10.5256/f1000research.152096.r183727

**Published:** 2023-07-11

**Authors:** Qijie Guan

**Affiliations:** 1 Department of Biology, University of Mississippi, University Park, Mississippi, USA

Species name *M. crystallinum *should be in italics, C3 should be C _3_.

 I have no further comments to make.

Is the work clearly and accurately presented and does it cite the current literature?

Yes

If applicable, is the statistical analysis and its interpretation appropriate?

Yes

Are all the source data underlying the results available to ensure full reproducibility?

Yes

Is the study design appropriate and is the work technically sound?

Yes

Are the conclusions drawn adequately supported by the results?

Yes

Are sufficient details of methods and analysis provided to allow replication by others?

Yes

Reviewer Expertise:

Bioinformatics, C3 to CAM transition, omics

I confirm that I have read this submission and believe that I have an appropriate level of expertise to confirm that it is of an acceptable scientific standard.

# Response

**Published:** 2023-07-14

**Authors:** Ryoma Sato

**Affiliations:** Kyushu University, Fukuoka, Fukuoka, Japan

Species name M. crystallinum should be in italics, C3 should be C _3_.

 ⇒ Thank you for your suggestion. I've adjusted the formatting of "M. crystallinum" from Roman to Italic and moved the "3" in "C3" to the lower right, as shown below:

 We succeeded in assembling the *M. crystallinum* genome using Illumina PE reads, characterizing the genome, and identifying the potential gene, non-transcriptional and translational regions, and repetitive sequences.

 The first released available genome of the common ice plant ( *Mesembryanthemum crystallinum* L.) extended the research region on salt tolerance, C _3_-CAM photosynthetic conversion, and halophilism.

 <” Conclusion” in Abstract>

 The information that we characterized is useful for elucidation of the mechanism of growth promotion under salinity and reversible conversion of the photosynthetic type from C _3_ to Crassulacean Acid Metabolism (CAM).

 Our results revealed that salt tolerance increases with growth, and C _3_-CAM photosynthetic conversion in the presence of NaCl is probably controlled by both protein-coding genes and potential genomic factors, including transposable elements, tRNAs, miRNAs, and protein kinases.

 Protocol.io: Methods in “The first released available genome of the common ice plant ( *Mesembryanthemum crystallinum* L.) extended the research region on salt tolerance, C _3_-CAM photosynthetic conversion, and halophism” V.1.

 Kong W, Yoo MJ, Zhu D, et al.: Molecular changes in *Mesembryanthemum crystallinum* guard cells underlying the C _3_ to CAM transition. *Plant Mol. Biol.* 2020;103(6):653–667. 32468353 10.1007/s11103-020-01016-9

 Sato R, Kondo Y, Agarie S: Methods in “the first released available genome of the common ice plant ( *Mesembryanthemum crystallinum* L.) extended the research region on salt tolerance, C _3_-CAM photosynthetic conversion, and halophism”. *protocols.io.* 2023a. 10.17504/protocols.io.6qpvr4qdogmk/v1

 Shen S, Li N, Wang Y, et al.: High-quality ice plant reference genome analysis provides insights into genome evolution and allows exploration of genes involved in the transition from C _3_ to CAM pathways. *Plant Biotechnol. J.* 2022;20(11):2107–2122. 35838009 10.1111/pbi.13892 PMC9616530

 Winter K, Ltittge U, Winter E, et al.: Seasonal shift from C _3_ photosynthesis to crassulacean acid metabolism in *Mesembryanthemum crystallinum* growing in its natural environment. *Oecologia (Berl).* 1978;34:225–237. 28309551 10.1007/BF00345168

 Yuan G, Hassan MM, Liu D, et al.: Biosystems design to accelerate C _3_-to-CAM progression. *BioDesign Res.* 2020;2020:3686791. 10.34133/2020/3686791

 There is no new figure, dataset, and change to author names or affiliation required.

# Reviewer report: Reviewer response for version 1

**DOI:** 10.5256/f1000research.142683.r178334

**Published:** 2023-06-23

**Authors:** Qijie Guan

**Affiliations:** 1 Department of Biology, University of Mississippi, University Park, Mississippi, USA

This study is of significant importance, and the authors have utilized the best genome assembly platform for shotgun sequencing. However, there are a few minor concerns that need to be addressed:

1. In the sample materials, it is mentioned that the ice plants were treated with 0.3% (w/w) NaCl for 5 weeks. Please double-check the unit, should it be w/v (weight/volume) instead of w/w (weight/weight)? Additionally, it would be helpful if the authors could provide information about the nutrient content in OAT1 House No.1 and No.2. Most importantly, the authors should clarify the CAM status of the ice plant before sampling.
2. Although the author compared the ice plant genome against several other plant species, no CAM plants or C4 plants were included. It is recommended that the authors consider adding pineapple to the comparison.
3. The authors mentioned the release date of the SILVA database as 2020.08. It would be preferable if the authors could change the version of the database to its standard name, SILVA 138.1.

Is the work clearly and accurately presented and does it cite the current literature?

Yes

If applicable, is the statistical analysis and its interpretation appropriate?

Yes

Are all the source data underlying the results available to ensure full reproducibility?

Yes

Is the study design appropriate and is the work technically sound?

Yes

Are the conclusions drawn adequately supported by the results?

Yes

Are sufficient details of methods and analysis provided to allow replication by others?

Yes

Reviewer Expertise:

Bioinformatics, C3 to CAM transition, omics

I confirm that I have read this submission and believe that I have an appropriate level of expertise to confirm that it is of an acceptable scientific standard, however I have significant reservations, as outlined above.

# Response

**Published:** 2023-06-24

**Authors:** Ryoma Sato

**Affiliations:** Kyushu University, Fukuoka, Fukuoka, Japan

Thank you for your suggestions.

- *In the sample materials, it is mentioned that the ice plants were treated with 0.3% (w/w) NaCl for 5 weeks. Please double-check the unit, should it be w/v (weight/volume) instead of w/w (weight/weight)? In the sample materials, it is mentioned that the ice plants were treated with 0.3% (w/w) NaCl for 5 weeks. Please double-check the unit, should it be w/v (weight/volume) instead of w/w (weight/weight)?*

Yes, as you suggested, the notation should be w/v (weight/volume), but to adopt a more general notation, we have converted the concentration of NaCl from w/v to molar concentration as follows:

 < “Plant materials and growth conditions” in Methods >

 The plants were treated with the solution including 51 mM NaCl for two weeks.

- *Additionally, it would be helpful if the authors could provide information about the nutrient content in OAT1 House No.1 and No.2.*

The expression on compositions of OAT House No. 1 and No. 2 have been changed as follows:

 < “Plant materials and growth conditions” in Methods >

 The plants were irrigated with a nutrient solution of 1.5 g L -1 OAT House No. 1, containing primary nutrients including 10% Nitrogen (1.5% as ammoniacal nitrogen and 8.2% as nitrate nitrogen), 8.0% water-soluble phosphoric acid, 27% water-soluble potassium, 4.0% water-soluble magnesium, 0.10% water-soluble manganese, 0.10% water-soluble boron, 0.18% iron, 2.0 × 10 ^-3^% copper, 6.0 × 10 ^-3^% zinc, and 2.0 × 10 ^-3^% molybdenum, in addition to 1.0 g L ^-1^ No. 2, comprising 11% nitrogen and 23% lime (OAT Agrio Co., Ltd., Tokyo, Japan) in a greenhouse at Kyushu University for five weeks.

- *Most importantly, the authors should clarify the CAM status of the ice plant before sampling.*

In terms of CAM status, I think this is not necessary. The objective of this paper is to decode the genome sequence and we did not perform gene expression analysis, such as RNA-Seq analysis. Also, this is a genome analysis, so we used untreated plants. Therefore, we did not measure the internal pH of the ice plant's leaves.

- *Although the author compared the ice plant genome against several other plant species, no CAM plants or C4 plants were included. It is recommended that the authors consider adding pineapple to the comparison.*

Thank you for your recommendation. The title of this paper includes the term “CAM”, but it did not mean that we will elucidate factors associated with CAM induction. The word “CAM” used in the introduction and elsewhere was to describe one of the characteristics of the common ice plant like the following six kinds of sentences.

 The information that we characterized is useful for elucidation of the mechanism of growth promotion under salinity and reversible conversion of the photosynthetic type from C3 to Crassulacean Acid Metabolism ( **CAM**).

 Also, it converts its photosynthetic mode from C3 to Crassulacean Acid Metabolism ( **CAM**) under severe salt stress and drought stress (Adams *et al*. 1998).

 These days, the comparative analysis of genome information focused on **CAM**-related genes in the common ice plants has been reported (Shen *et al*. 2022), but this genome resource is not easy to use.

*M. crystallinum* is utilized as a model plant for investigating halophilism, salt tolerance, and **CAM** photosynthesis.

 Several miRNA-targeting transcription factors were associated with salt tolerance ( *HLH*, *SPL*, *HD-ZIP*) (Shen *et al. *2019; Wang *et al. *2019; Wang *et al. *2021) or **CAM **photosynthesis ( *WRKY*, *AP2*, *MYB*, *NAC*) (Amin *et al. *2019; Yuan *et al. *2020; Shah *et al. *2021).

 The first five sections, as explained earlier, contain the term “CAM” solely for the purpose of describing the physiological traits of the ice plant. The sixth section suggests that the discovery of miRNA could serve as a catalyst to advance research on salt tolerance and CAM. As such, the term CAM is seldom mentioned in this paper, and wherever the word is used, it is only for mere description or hinting at potential advancements in future research. Overall, the purpose of this study is to elucidate the characteristics of the ice plant's genome, so we did not focus on CAM this time. Instead, we are currently progressing with functional genomics analysis related to CAM and other traits using a full-length genome sequence with PacBio Sequel II and publicly available Hi-C data. Soon, we will submit a paper revealing the impact of the genome on the C3-CAM transition mechanism of the ice plant and the growth promotion mechanism under the presence of NaCl.

- *The authors mentioned the release date of the SILVA database as 2020.08. It would be preferable if the authors could change the version of the database to its standard name, SILVA 138.1.*

Thank you for the suggestion. I changed the version number as follows:

 <” Phylogenetic tree creation among multiple plant species using 18S ribosomal DNA sequences” in Methods >

 …were selected using the SILVA 138.1 database (Release. 2020-08; Pruesse *et al*. 2007).

 The above is my reply. I appreciate your understanding and assistance in this matter despite your busy schedule.
